# Contemporary Precision Stratification and Prognostic Features of Primary Gliomas in a Southern Chinese Population

**DOI:** 10.34133/research.1014

**Published:** 2025-12-09

**Authors:** Shanqiang Qu, Zhi Ye, Qiuming Pan, Haiyan Xu, Hongrui Li, Junxi Wang, Xin Zhang, Yilamujiang Ainiwan, Luyao Wang, Guozhong Yi, Jinfeng Lin, Zhiyong Li, Xiaoxia Zheng, Tingping Xie, Yudi Huang, Tao Liu, Xi’an Zhang, Songtao Qi, Guanglong Huang

**Affiliations:** ^1^ Department of Neurosurgery, Nanfang Hospital, Southern Medical University, Guangzhou, Guangdong 510515, People’s Republic of China.; ^2^ Nanfang Glioma Center, Nanfang Hospital, Southern Medical University, Guangzhou, Guangdong 510515, People’s Republic of China.; ^3^ Institute of Brain Disease, Nanfang Hospital, Southern Medical University, Guangzhou, Guangdong 510515, People’s Republic of China.; ^4^ Laboratory for Precision Neurosurgery, Nanfang Hospital, Southern Medical University, Guangzhou, Guangdong 510515, People’s Republic of China.; ^5^ The First Clinical School of Medicine, Southern Medical University, Guangzhou, Guangdong 510515, People’s Republic of China.; ^6^ Department of Laboratory Medicine, Guangdong Provincial Key Laboratory of Precision Medical Diagnostics, Guangdong Engineering and Technology Research Center for Rapid Diagnostic Biosensors, Guangdong Provincial Key Laboratory of Single Cell Technology and Application, Nanfang Hospital, Southern Medical University, Guangzhou, Guangdong 510515, People’s Republic of China.

## Abstract

With the significant transformation in the classification, risk stratification, and therapy standards for gliomas in recent years, we sought to reintegrate clinical data, whole-exome sequencing data, and magnetic resonance imaging data from glioma patients to further analyze their impact on overall survival. We identified 798 primary gliomas: 355 glioblastomas, 179 *IDH1/2*-mutant astrocytomas, 135 oligodendrogliomas, and 129 other *IDH1/2*-wild-type gliomas. Kaplan–Meier analysis revealed that our cohort showed significantly prolonged survival compared to the CGGA/TCGA cohorts (median: 85.2, 60.4, and 50.5 months; *P* < 0.0001). Molecular reclassification criteria yielded altered final histopathologic classification for 23.7% of gliomas. Molecular alterations differ among glioma subtypes. Among the 5 tumorigenic pathways analyzed, glioblastomas exhibited the highest average number of activated pathways (mean: 2.17), followed by astrocytomas (mean: 1.40) and oligodendrogliomas (mean: 0.42). In one glioma subtype, upstream and downstream gene activations in the same pathway are mutually exclusive. In this large-scale Chinese cohort, we first confirmed a strong link between tumor location and molecular subtype: Frontal gliomas had *IDH1/2* mutations in 63.5% of cases, while temporal (80.3%) and thalamic/basal ganglia gliomas (90.4%) were predominantly *IDH1/2*-wild-type. Age stratification confirmed these patterns: 74.7% of frontal gliomas in younger patients (<46 years) had *IDH1/2* mutations versus 91.4% of temporal and 100% of thalamic/basal ganglia tumors in older patients (≥46 years) being *IDH1/2*-wild-type. Contemporary molecular criteria modified diagnoses in ~25% of cases. Contemporary glioma cohorts showed prolonged survival outcomes compared to historical cohorts. An association between anatomic localization and molecular subtypes was also established in this Chinese glioma cohort.

## Introduction

Gliomas, the most common primary intracranial malignancies, are characterized by high recurrence and disability rates that substantially deteriorate patients’ quality of life [1]. Among the gliomas, glioblastoma (GBM) is the most aggressive subtype, with a median survival time of <15 months despite standard treatment involving surgical resection combined with chemoradiotherapy [2,3]. Moreover, there is a lack of effective secondary therapies for recurrent gliomas, resulting in failure to prolong the overall survival (OS) of patients. The marked heterogeneity in the clinical outcomes of glioma patients highlights the limitations of traditional histopathological classification in meeting the demands for personalized therapy in the precision medicine era.

The completion of the Human Genome Project and advancements in high-throughput sequencing have revolutionized our understanding of glioma pathogenesis through systematic investigations utilizing databases such as The Cancer Genome Atlas (TCGA). On the basis of these developments, the 2021 World Health Organization (WHO) classification of central nervous system (CNS) tumors now incorporates molecular markers, including *IDH1/2* mutations and 1p/19q co-deletion, into diagnostic criteria [4]. Notably, some of the glioma cases might show a discrepancy between pathologic and molecular findings [4]. Although whole-exome sequencing (WES) has revealed critical molecular associations such as *IDH*-*TERTp* mutual exclusivity and *EGFR*-*PTEN* co-mutations, the clinical implications of genomic alterations depend substantially on the tumor type [5]. For instance, identical mutations may lead to divergent prognostic outcomes across cancer types [6–8]. The 2021 WHO Classification of Central Nervous System Tumors achieves refined risk stratification by elevating the prognostic weight of molecular markers (e.g., CDKN2A/B homozygous deletion directly defining grading) and incorporating novel molecularly defined tumor entities. This marks a paradigm shift from histology-based to genetics-driven diagnostic and grading frameworks. Furthermore, distinct variations exist between Chinese and Western glioma cohorts regarding age of onset, molecular profiles, clinical management, and survival outcomes. For instance, the higher prevalence of *IDH* mutations and younger patient demographics in China contrast with the predominance of elderly-onset GBM in the United States.

In our study, we aim to curate and utilize a large-scale, clinically heterogeneous glioma cohort from southern China. By reclassifying patients according to modern WHO CNS5 classification criteria, we will investigate (a) significant clinical and molecular disparities between contemporary versus historical cohorts, (b) distinct mutational signatures across glioma subtypes and magnetic resonance imaging (MRI) features, and (c) subtype-dependent determinants of OS.

## Results

### Demographic characteristics of 798 patients with primary gliomas

**Fig. 1.**
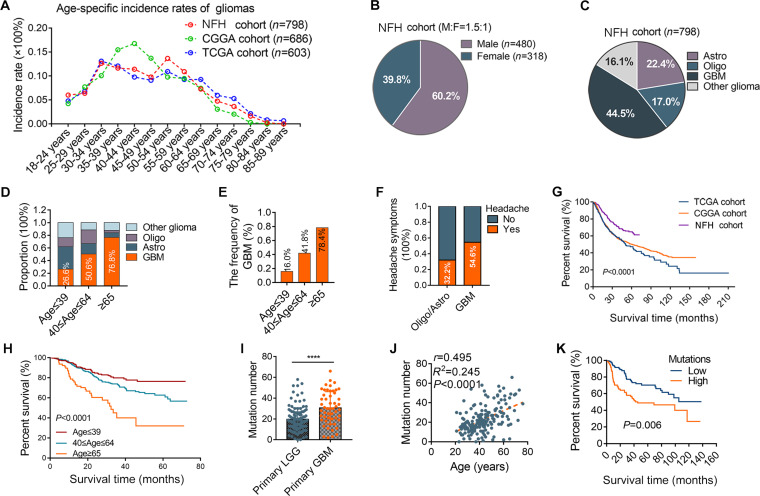
Demographic characteristics of primary glioma cohorts. (A) Bimodal age distribution of 798 primary glioma patients (NFH cohort), with peaks at 30 to 34 and 50 to 54 years. (B) Male predominance across cohorts. (C) Patient proportion in each subtypes including glioblastoma, *IDH1/2*-mutant astrocytoma, oligodendroglioma, and other *IDH1/2*-wild-type gliomas. (D) Glioblastoma prevalence escalates with age (*n* = 798). (E) Validation of age-dependent subtype shifts in CGGA cohorts (*n* = 686). (F) Symptom profiles: higher headache prevalence in glioblastoma versus low-grade glioma (LGG). (G) Kaplan–Meier survival analysis showing prolonged survival in the NFH cohort compared to CGGA/TCGA cohorts (log-rank test, *P* < 0.0001; NFH, *n* = 798; CGGA, *n* = 686; TCGA, *n* = 603). (H) Age-stratified survival within the NFH cohort (*n* = 798). Survival differences were compared using the log-rank test (*P* < 0.0001). (I) TMB is elevated in glioblastoma versus LGG in CGGA cohort. (J) Positive correlation between TMB and age in CGGA cohort. (K) Higher TMB predicts worse prognosis in CGGA cohort.

We identified 798 unique patients with primary gliomas in Nanfang Hospital of Southern Medical University (NFH) from January 2019 to January 2024. The clinicopathologic characteristics are summarized in Table S1. The cohort exhibited a bimodal age distribution (peaks at 30 to 34 and 50 to 54 years; Fig. 1A), consistent with the TCGA data. Male predominance was observed (60.2%; male-to-female ratio, 1.5:1; Fig. 1B), which was validated by the Chinese Glioma Genome Atlas (CGGA) cohort (*n* = 686) and TCGA cohort (*n* = 603) (Fig. S1). Our cohort included 355 cases of GBM (44.5%), 179 cases of astrocytoma (22.4%), 135 cases of oligodendroglioma (17.0%), and 129 cases of other *IDH1/2*-wild-type gliomas (16.1%) (Fig. 1C), all classified according to the WHO classification of tumors of the CNS 2021 guidelines. The prevalence of GBM increased with age, constituting 26.6% (78/293) of cases in patients ≤39 years, 50.6% (214/423) of cases in patients with age 40 to 64 years, and 76.8% (63/82) of cases in patients ≥65 years (Fig. 1D). This age-dependent increase in GBM prevalence was also noted in the CGGA and TCGA cohorts (Fig. 1E and Fig. S2). Clinical symptom analysis revealed headache in 54.6% of GBM patients compared to 32.2% of patients with low-grade glioma (Fig. 1F). In the Kaplan–Meier analysis of OS, the NFH cohort showed significantly prolonged survival compared to the CGGA and TCGA cohorts (*P* < 0.0001; Fig. 1G), and stratification analyses further validated this trend (Fig. S3). Additionally, the 3 cohorts were matched using propensity score matching analysis for further statistical comparisons (Fig. S3). Stratification by age in the NFH cohort further demonstrated reduced survival probabilities with advanced age (*P* < 0.0001; Fig. 1H). This result was also confirmed in the CGGA and TCGA cohorts (*P* < 0.0001 and *P* < 0.0001, respectively; Fig. S4). Univariate and multivariate Cox regression analyses were performed to estimate independent prognostic factors for patients, and results are shown in Table S2.

GBM is a polygenic disorder, with mutations, amplifications, and deletions in multiple genes [9]. A total of 4,003 mutations were detected in 175 samples from CGGA cohort (median: 20 mutations per case [range: 0 to 66]). The peak age of onset was approximately 40 years in patients with *IDH1/2-*mutant gliomas and approximately 50 years in patients with *IDH1/2*-wild-type gliomas (Fig. S5). GBM exhibited a higher tumor mutation burden (TMB) (median, 30 mutations per case) compared to low-grade glioma (median, 18 mutations per case; *P* < 0.001; Fig. 1I). Mutation frequency increased with age, and a positive correlation was observed between TMB and age [*r* = 0.495; 95% confidence interval (CI), 0.375 to 0.600; *P* < 0.0001; Fig. 1J]. OS analysis revealed that patients with a higher TMB had significantly worse prognosis (hazard ratio = 1.895 [1.201 to 2.989]; *P* = 0.006; Fig. 1K).

### Contemporary molecular features refine histopathological diagnoses

**Fig. 2.**
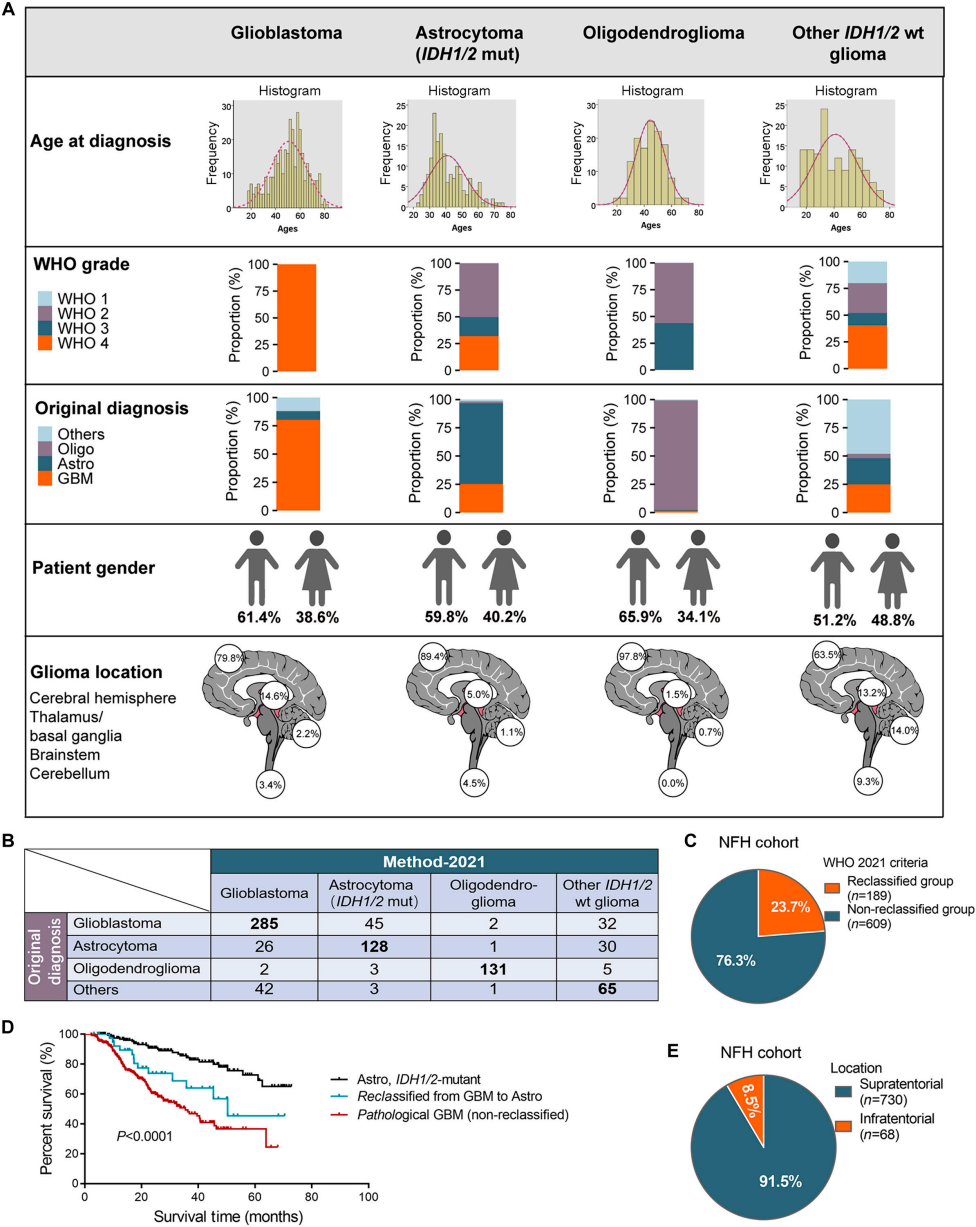
Diagnostic reclassification and survival outcomes by molecular profiling. (A) Summary of the pooled NFH glioma cohort (*n* = 798). (B) Diagnostic concordance between original diagnoses and molecular classification (method-2021). (C) Molecular profiling revised diagnoses in 23.7% of cases. (D) Survival prognosis comparison between non-reclassified GBM patients (*n* = 285), astrocytoma patients reclassified from GBM (*n* = 45), and astrocytoma with *IDH1/2*-mutant (*n* = 179). Survival differences were compared using the log-rank test. (E) Anatomical distribution: supratentorial tumors predominated over infratentorial.

We stratified 798 primary gliomas into 4 molecular subgroups: GBM, *IDH1/2*-mutant astrocytoma, oligodendroglioma, and other *IDH1/2*-wild-type gliomas. According to age distribution, GBM patients exhibited the oldest median age at diagnosis (52 years), followed by patients with *IDH1/2*-wild-type glioma (39 years), patients with oligodendroglioma (44 years), and patients with *IDH1/2*-mutant astrocytoma (38 years; Fig. 2A). Consistent with broader cohort trends, male predominance persisted across all glioma subtypes (male-to-female ratio range, 1.1 to 1.9:1; Fig. 2A), although the difference in the sex hormone-dependent incidence rate remained speculative [10]. Molecular classification significantly refined glioma subtypes from the original histopathological diagnoses. While 80.3% (285/355) of GBM cases retained their original histopathological designation, *IDH1/2*-mutant astrocytoma cases demonstrated substantial diagnostic heterogeneity, with 71.5% (128/179) of cases initially classified as astrocytoma, 25.1% (45/179) of cases as GBM, 1.7% (3/179) of cases as oligodendroglioma, and 1.7% (3/179) of cases as other glioma subtypes (Fig. 2B). In contrast, oligodendroglioma cases showed near-perfect concordance (97.0%) with the original diagnoses, whereas other *IDH1/2*-wild-type glioma cases were frequently misclassified as GBM cases (24.8%) or astrocytoma cases (23.3%) prior to molecular analysis (Fig. 2B). Notably, molecular profiling revised the diagnoses in 23.7% (189/798) of cases, highlighting the limitations of conducting only histopathological examination (Fig. 2C). The Kaplan–Meier survival curves showed that patients reclassified from GBM to *IDH1/2*-mutant astrocytoma (*n* = 45) had significantly better survival than those with *IDH1/2*-wild-type GBM (*n* = 285), but worse survival than patients with *IDH1/2*-mutant astrocytoma (*n* = 179) (*P* < 0.0001; Fig. 2D). This further indicates a statistically significantly survival disparity between patients reclassified from GBM to *IDH*-mutant astrocytoma under contemporary molecular criteria and those retaining the GBM diagnoses.

Additionally, the number of gliomas with supratentorial occurrence (91.5%) was considerably higher than that with infratentorial occurrence (8.5%; Fig. 2E). The 4 molecular subgroups exhibited further differences in their anatomical localization patterns: *IDH1/2*-mutant gliomas (astrocytomas and oligodendrogliomas) predominantly occupied the cerebral hemispheres (89.4% and 97.8%, respectively), with minimal cerebellar involvement (1.1% and 0.7%, respectively). However, the proportion of *IDH1/2*-wild-type gliomas in the thalamus–basal ganglia (13.9%) and the infratentorial region (14.5%) was significantly higher than that of *IDH1/2-*mutant gliomas (3.3% and 3.2%, respectively).

### Mutational landscape of adult diffuse gliomas

**Fig. 3.**
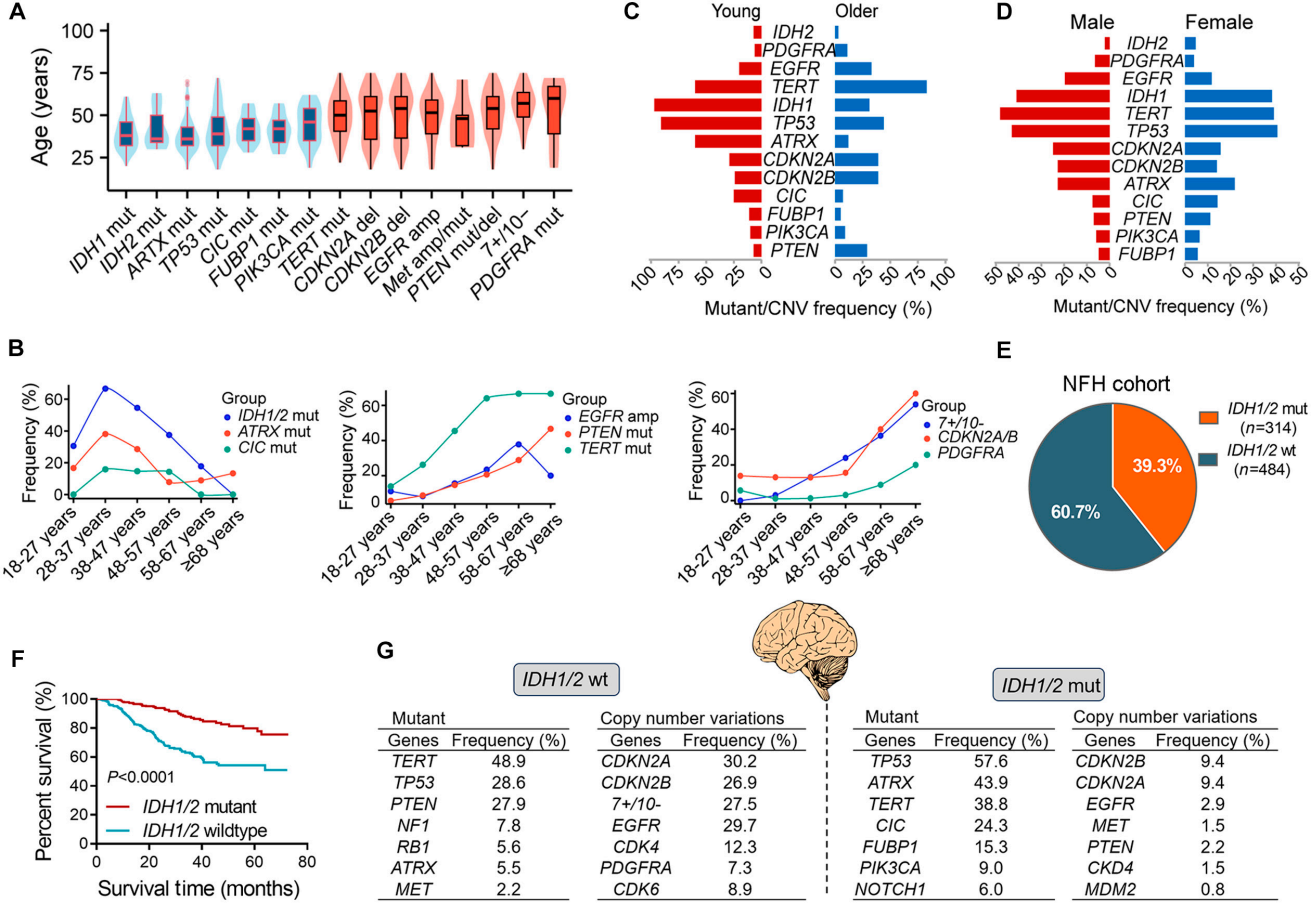
Age-associated mutational hierarchies and subtype-specific alterations. (A and B) Age-associated molecular alterations in glioma patients. (C and D) Molecular alterations exhibited distinct overall frequencies between younger and older groups, with minimal disparities observed across genders. (E) *IDH1/2* status distribution in NFH cohort. (F) Patients with *IDH1/2*-mutant have longer survival than those with *IDH1/2-*wild-type (log-rank test, *P* < 0.0001). (G) Distribution of common genetic mutations and copy number variations in *IDH1/2*-mutant versus *IDH1/2*-wild-type cohorts.

Our analysis confirmed that the age distribution of glioma patients is strongly linked to molecular subtypes. The genomic profile defining *IDH1/2*-mutant gliomas (e.g., *IDH1/2*, *ATRX*, and *TP53* mutations) was significantly enriched in younger patients (≤40 years), whereas the profile characteristic of *IDH1/2*-wild-type GBM (e.g., *TERTp* mutations, *EGFR* amplification, *PTEN* alterations, and chromosome 7+/10−) predominantly occurred in older patients (≥58 years) (Fig. 3A and B). Molecular alterations showed a significant difference between younger and older age groups (Fig. 3C), with minimal gender-based differences (Fig. 3D).

In our cohort, 314 patients (39.3%) had *IDH1/2-*mutant gliomas, while 484 (60.7%) had *IDH1/2*-wild-type gliomas (Fig. 3E). Table 1 revealed significant clinical and histopathological differences between the 2 groups. Clinically, *IDH1/2*-mutant gliomas were associated with lower headache incidence (32.2% versus 50.4% in *IDH1/2*-wild-type gliomas; *P* < 0.0001) but higher epilepsy prevalence (36.0% versus 16.9%; *P* < 0.001). Prognostically, patients with *IDH1/2*-mutant gliomas showed superior survival than those with *IDH1/2*-wild-type tumors (*P* < 0.0001; Fig. 3F). The 2 groups also showed a significant difference in genomic features. Table S2 depicts the validated somatic mutants from the exome sequencing of gliomas. In *IDH1/2*-mutant gliomas, the characteristic alterations included *IDH1* R132H, R132G, or R132S substitution and *IDH2* R172K or R172G substitution, with frequent co-occurrence of mutations in *TP53* (57.6%) and *ATRX* (43.9%) (Fig. 3G). These aberrations predominated in astrocytomas (*TP53*: 87.2%; *ATRX*: 68.6%), whereas oligodendrogliomas showed minimal *TP53* (9.4%) and *ATRX* (3.8%) alterations. Among *IDH1/2*-mutant gliomas, *TERTp* (38.8%), *CIC* (24.3%), and *FUBP1* (15.3%) alterations exhibited a subtype-specific distribution, with *CIC*/*FUBP1* mutations predominant in oligodendrogliomas (62.0%/39.2%) and rare (<2%) in non-oligodendroglial subtypes. Strikingly, 92% of oligodendrogliomas harbored active *TERTp* mutations, compared to 12.2% in astrocytomas, thus highlighting mutual exclusivity between *ATRX* and *TERTp* alterations. In *IDH1/2*-mutant gliomas, elevated *TERTp* mutation rates predominantly reflected oligodendroglioma molecular profiles (with 1p/19q co-deletion), whereas astrocytoma subtypes were characterized by higher *ATRX* mutation rates. Notably, in *IDH1/2*-mutant glioma, *CDKN2A/B* deletion and *EGFR* amplifications were rare but confined to high-grade gliomas. In *IDH1/2*-wild-type gliomas, high frequencies of *TERTp* mutations (48.9%), *EGFR* amplifications (29.7%), *PTEN* alterations (27.9%), and chromosomal 7+/10− alterations (27.5%) correlated with advanced age and poor prognosis (Fig. 3F). Notably, chromosomal 7+/10− alterations (encompassing *EGFR* and *PTEN* loci) were rare in *IDH1/2*-mutant gliomas (1.0% in high-grade gliomas). While *TP53* mutations occurred in 28.6% of GBM cases, this frequency was significantly lower than that in *IDH1/2*-mutant astrocytoma cases (87.2%). Aggressive subsets within wild-type gliomas were further defined by *CDKN2A/B* deletion (26.9%) and *NF1* mutation (7.8%).

**Table 1.** Clinical characteristics of the sample set arranged by IDH1/2 status

Feature	*IDH1/2* mut (*n* = 314)	*IDH1/2* wt (*n* = 484)	*P* value
**Clinical**			
Gender			
Male	196	284	0.291
Female	118	200	
Body mass index			
BMI < 18.5	13	39	0.043
18.5 ≤ BMI < 25	209	277	
BMI ≥ 25	86	125	
NA	6	43	
KPS at diagnosis			
≥70	305	448	0.006
<70	9	36	
Headache symptom at diagnosis
Yes	101	244	<0.001
No	213	240	
Epilepsy at diagnosis			
Yes	113	82	<0.001
No	201	402	
**Histopathology**			
Histology (*n*)			
Glioblastoma	47	318	<0.001
Astrocytoma	129	56	
Oligodendroglioma	134	7	
Others	4	107	
WHO grade (*n*)			
WHO I	0	26	<0.001
WHO II	166	36	
WHO III	91	15	
WHO IV	57	407	
Ki-67 index			
Ki-67 < 5%	114	86	<0.001
5% ≤ Ki-67 ≤ 20%	137	137	
20% < Ki-67 ≤ 50%	51	178	
Ki-67 > 50%	12	83	
**Molecular**			
*1p/19q* status			
Codeletion	135	6	<0.001
Non-codeletion	179	421	
NA	0	57	
*MGMT*p methylation			
Yes	111	81	<0.001
No	38	114	
NA	165	289	

### Clinical characteristics, molecular alterations, and outcome vary across glioma subtypes

**Fig. 4.**
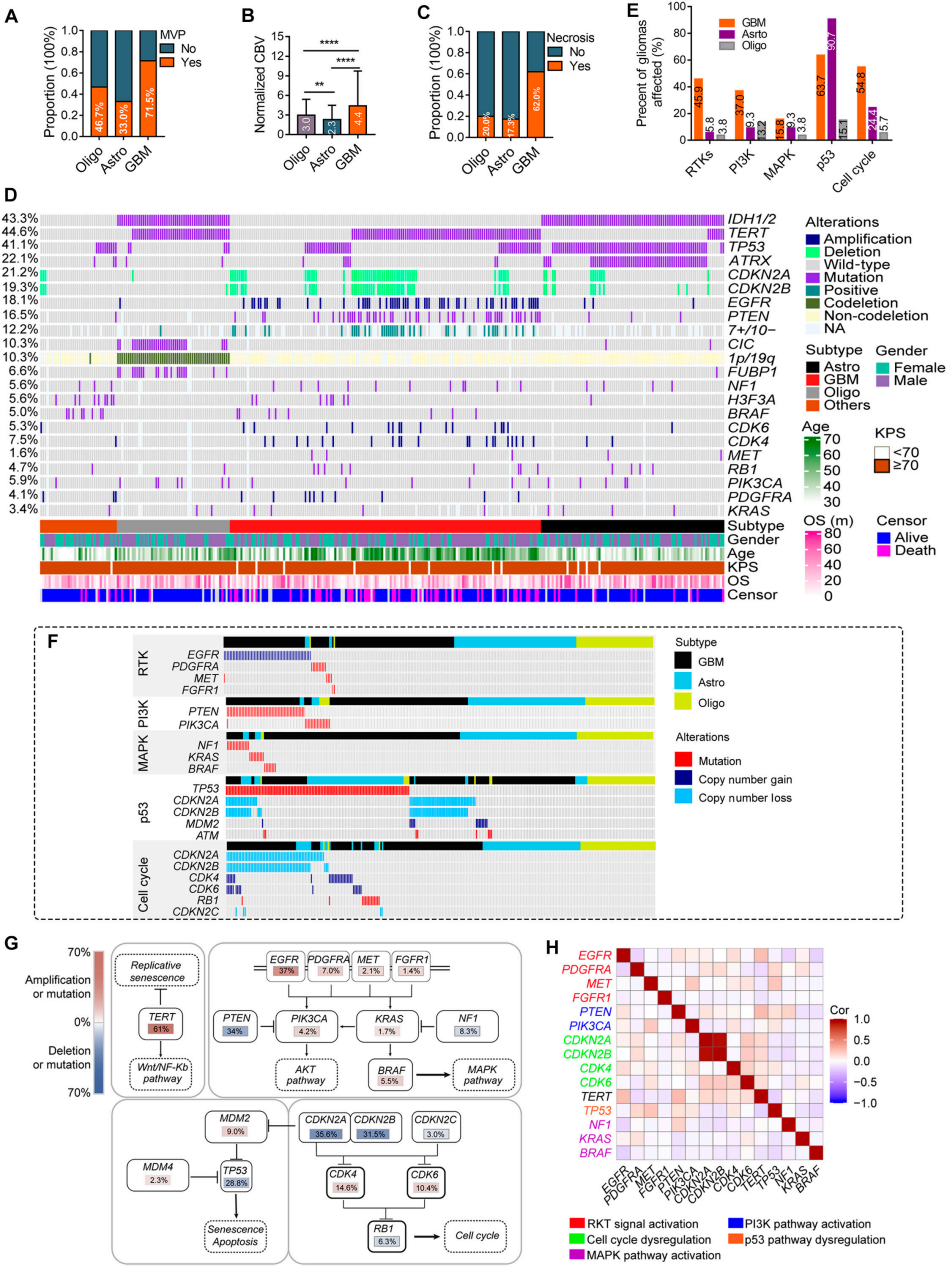
Clinicopathologic and molecular heterogeneity across subtypes. (A to C) Comparative analysis of MVP, nCBV, and tumor necrosis prevalence among GBM, astrocytoma, and oligodendroglioma. **P < 0.01; ****P < 0.0001 (Mann–Whitney *U* test). (D) Mutational signatures and CNVs of gliomas stratified by tumor subtypes. (E) Proportion of gliomas with an affected tumorigenic pathway: RTK, PI3K, MAPK, p53, and cell cycle. (F) Molecular alterations within the same tumorigenic pathway were frequently mutually exclusive in glioma. (G) Mutation frequencies of signature genes across key signaling pathways in GBM. (H) Co-occurrence analysis of common genetic alterations in GBM.

We further stratified 798 primary gliomas into 4 molecular subgroups. Clinical features of these subgroups are summarized in Table 2. The median age of the cohort was 46 years (range: 18 to 81 years). Headache (54.6%) and elevated intracranial pressure (50.1%) were more common in patients with GBM, whereas the prevalence of epilepsy was higher in patients with astrocytoma (33.5%) and patients with oligodendroglioma (39.3%; *P* < 0.0001). Microvascular proliferation (MVP) was markedly higher in patients with GBM (71.5%) than in patients with oligodendroglioma (46.7%) or astrocytoma (33.0%) (Fig. 4A). Perfusion imaging revealed differences in normalized cerebral blood volume (nCBV): GBM exhibited the highest vascularity (nCBV = 4.4) compared to astrocytomas (nCBV = 2.3; *P* < 0.0001; Fig. 4B). Tumor necrosis was most prevalent in GBM (62.0%), followed by astrocytomas (17.3%) and oligodendrogliomas (20.0%) (Fig. 4C).

**Table 2.** Patient characteristics for glioma subtypes

Tumor type	Glioblastoma	Astrocytoma (*IDH1/2* mut)	Oligodendroglioma	Other *IDH1/2* wt glioma
Patient (*n*, %)	355 (44.5%)	179 (22.4%)	135(16.9%)	129 (16.2%)
Gender				
Male	218	107	89	66
Female	137	72	46	63
Body mass index				
BMI < 18.5	28	8	5	11
18.5 ≤ BMI < 25	205	119	90	72
BMI ≥ 25	88	51	35	37
NA	34	1	5	9
Karnofsky performance status at diagnosis				
≥70	325	174	131	123
<70	30	5	4	6
Headache symptom at diagnosis				
Yes	194	58	43	50
No	161	121	92	79
Epilepsy at diagnosis				
Yes	50	60	53	32
No	305	119	82	97
Tumor location				
Supratentorial	331	168	133	98
Posterior fossa	24	11	2	31
Side of tumor				
Left side	141	79	52	56
Right side	174	84	71	44
Both side	19	9	12	10
Middle	21	8	0	19
Contrast-enhanced T1-weighted imaging				
Yes	324	97	70	86
No	29	82	65	43
NA	2	0	0	0
Increased intracranial pressure at diagnosis				
Yes	178	67	28	35
No	177	112	107	94
Extent of resection				
Biopsy	1	0	0	1
Partial resection	25	19	10	21
Subtotal and total resection	329	160	125	107
Original diagnosis				
Glioblastoma	285	45	2	32
Astrocytoma	26	128	1	30
Oligodendroglioma	2	3	131	5
Others	42	3	1	62
*IDH1/2* status				
Mutant	0	179	135	0
Wild-type	355	0	0	129
*1p/19q* status				
Codeletion	4	0	135	2
Non-codeletion	311	179	0	110
NA	40	0	0	17

Canonical molecular alterations also showed various trends across the glioma subtypes (Fig. 4D). For example, whole chromosomal 7+/10− alterations occurred in 26% of patients with GBM, whereas partial chromosomal 7+/10− alterations occurred in additional 13% of patients with GBM. Whole chromosomal 7+/10− alterations in non-GBM subtypes were restricted to grade 3 to 4 tumors. *TERTp* mutations (61%) and *EGFR* amplifications (34%) were frequent in patients with GBM. *CDKN2A/B* deletions occurred in 32% of GBM cases, with partial *CDKN2A/B* deletions in an additional 4% of GBM cases. *IDH1/2*-mutant astrocytomas rarely exhibited *EGFR* amplifications (3%), which were confined to high-grade tumors. Oligodendrogliomas showed high *TERTp* (87%) and *CIC* (49%) mutation rates.

Analysis of 5 tumorigenic pathways, namely, receptor tyrosine kinase (RTK), phosphatidylinositol 3-kinase (PI3K), mitogen-activated protein kinase (MAPK), p53, and cell cycle, revealed divergent activation patterns. GBM exhibited the highest pathway co-alterations (mean: 2.17 pathways affected; median: 2), followed by *IDH1/2*-mutant astrocytomas (mean: 1.40; median: 1) and oligodendrogliomas (mean: 0.42; median: 1) (Fig. 4E). A total of 67.8% of GBM cases harbored ≥2 pathway alterations, predominantly p53 (63.7%) and cell cycle (54.8%) activation, with 52.1% of cases showing co-occurrence. In contrast, *IDH1/2*-mutant astrocytoma cases demonstrated high p53 (90.7%) and low cell cycle (24.4%) activation, while oligodendroglioma cases showed minimal activation of both pathways (15.1% and 5.7%, respectively). However, oligodendrogliomas exhibit preferential activation of the p53 and PI3K signaling pathways. Notably, gliomas rarely exhibited multiple molecular alterations in individual pathways (Fig. 4F). For example, RTK alterations (e.g., *EGFR, PDGFRA*, *MET*, and *FGFR1*) showed limited co-occurrence (Fig. 4G and H). *EGFR* amplification (37%) dominated RTK alterations. PI3K and MAPK pathway alterations were mutually exclusive: PI3K pathway co-alterations (e.g., *PIK3CA* and *PTEN*) occurred in 0.7% of GBM cases, while MAPK mutations (*NF1*, *BRAF*, and *KRAS*) were nearly mutually exclusive. In the p53 pathway, *TP53* mutations predominated in *IDH1/2*-mutant astrocytomas, whereas *CDKN2A/B* deletions were more common in GBM cases. Strikingly, *MET* mutations correlated positively with *PIK3CA* alterations (Fig. 4H).

In an analysis of chromosomal alterations across glioma subtypes (81 GBM, 48 *IDH1/2*-mutant astrocytomas, and 25 oligodendrogliomas), distinct genomic instability patterns were identified (Fig. S6). GBM cases showed chromosomal gains in chromosome 7 (36.0%); losses in chromosomes 10 (26.0%) and 9 (26%); and focal alterations, including *EGFR* (7p11.2) amplification (27.2%), *PTEN* (10q23) alterations (13.6%), and *CDKN2A/B* (9p21.3) deletions (22.2%). In *IDH1/2*-mutant astrocytomas, isolated 19q variations were identified in 19.0% of cases. These involved genes such as *NOTCH3* (19q13.12) or *CIC* (19q13.2). Recurrent 13q deletions (19.0%) involving *RB1* (13q14.2) lead to loss or dysfunction of the pRb protein. Oligodendrogliomas exhibited almost universal 1p/19q co-deletion (100%), with 13q deletions (28%), chromosome 7 amplifications (24%), and 11p amplifications (12%). The 1p/19q co-deletion involved *CIC* (1p36.23) and *FUBP1* (19q13.2).

### Integrated analysis of anatomic and molecular heterogeneity in gliomas

**Fig. 5.**
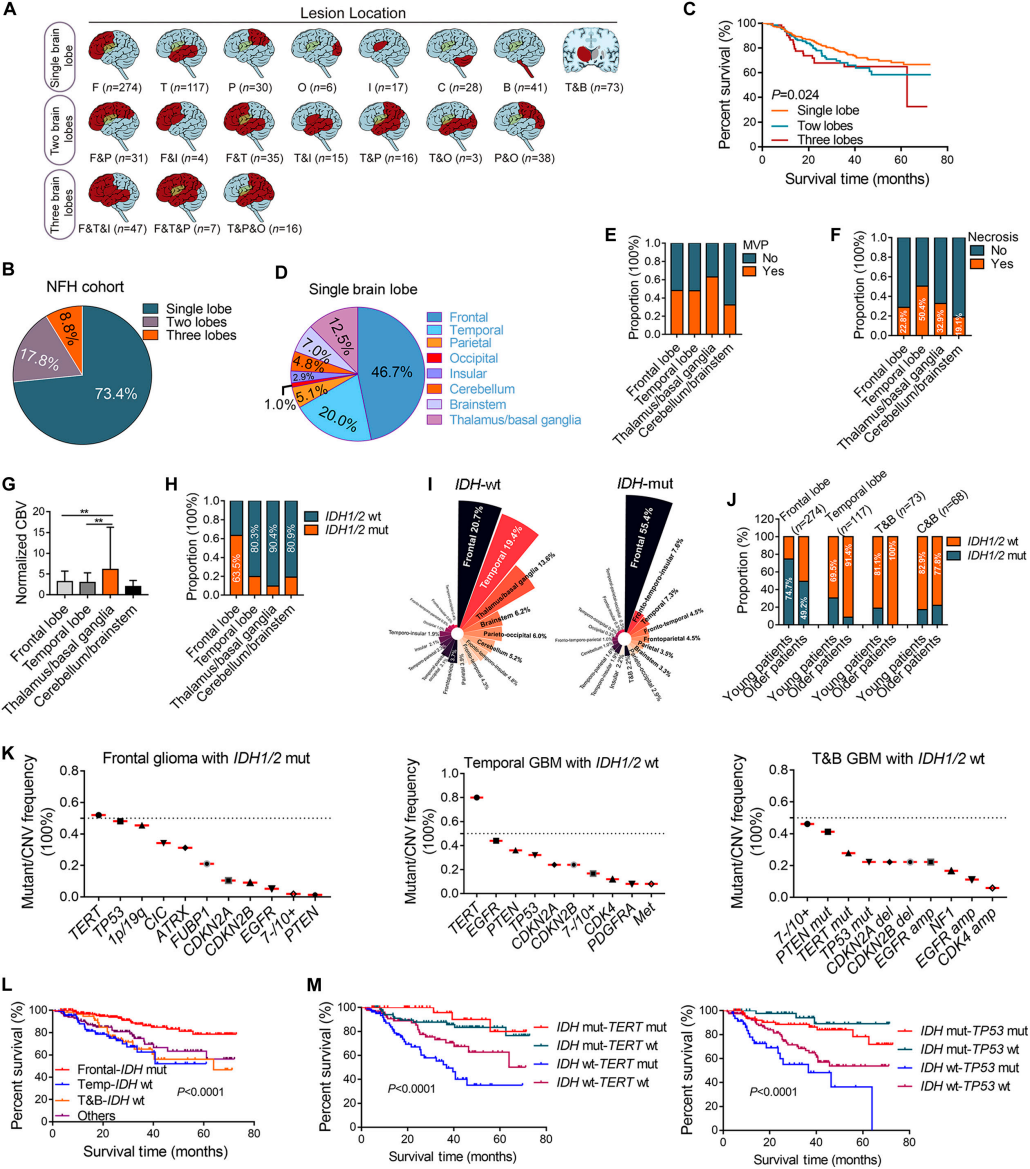
Anatomical–molecular correlations and prognostic implications. (A and B) Anatomic distribution of primary gliomas in 798 cases. (C) Patients with tumors involving a single lobe demonstrate significantly more favorable clinical outcomes compared to those with gliomas affecting 2 or more lobes (log-rank test, *P* = 0.024). (D) Anatomic distribution of single-lobe gliomas (*n* = 585). (E to G) Comparative analysis of MVP, nCBV, and tumor necrosis prevalence among gliomas in distinct lobes (*n* = 585). (H) Distribution of *IDH* status in gliomas across lobes. (I) Lobar distribution disparities between *IDH1/2-*mutant (*n* = 314) and *IDH1/2*-wild-type (*n* = 484) glioma subtypes. (J) Frontal lobe gliomas in younger patients are predominantly *IDH1/2*-mutant, whereas those in the temporal lobe or thalamic–basal ganglia region among older patients are primarily *IDH1/2*-wild-type. (K) Frequency of molecular alterations in gliomas with anatomic predilection. (L) Patients with *IDH1/2*-wild-type gliomas in the temporal lobe or thalamic–basal ganglia region demonstrate the poorest prognosis. (M) *TERTp* mutation serves as a favorable prognostic factor in *IDH1/2*-mutant gliomas, whereas it portends poor prognosis in *IDH1/2*-wild-type gliomas; conversely, *TP53* mutation consistently demonstrates adverse prognostic significance across all glioma subtypes. Survival differences were compared using the log-rank test.

Next, we further investigate the distribution of tumor location. Single-lobe involvement was observed in 586 patients (73.4%), while 142 (17.8%) and 70 (8.8%) patients had tumors spanning 2 or 3 lobes, respectively (Fig. 5A and B). Overall, survival analysis revealed better prognosis for patients with single-lobe tumors than for those with multilobar involvement (Fig. 5C). Among single-lobe tumors, the frontal lobe was the most frequent site of occurrence (46.7%), followed by the temporal lobe (20.0%) and the thalamic/basal ganglia region (12.5%) (Fig. 5D). Distinct pathologic features correlated with anatomic location: thalamic/basal ganglia gliomas exhibited prominent MVP (Fig. 5E), whereas temporal lobe tumors showed higher necrosis rates (Fig. 5F). Hemodynamic perfusion analysis further highlighted regional differences, with thalamic/basal ganglia tumors showing the highest perfusion levels (Fig. 5G).

Molecular profiling analysis revealed unique anatomic stratification of *IDH1/2* status. Frontal lobe gliomas harbored *IDH1/2* mutation in 63.5% of cases compared to 36.5% of cases with *IDH*1/2-wild-type tumors (Fig. 5H). Temporal lobe (80.3%) and thalamic/basal ganglia (90.4%) gliomas predominantly belonged to the *IDH1/2*-wild-type glioma subtype (Fig. 5H). Next, we stratified patients according to the *IDH1/2* mutation status and found that 55.4% of *IDH1/2*-mutant gliomas originated in the frontal lobe compared to 20.7% of *IDH1/2*-wild-type tumors (Fig. 5I). In contrast, *IDH1/2*-wild-type gliomas predominantly originated in the temporal lobe (19.4%) and thalamic/basal ganglia (13.6%) compared to *IDH1/2-*mutant tumors (7.3% and 2.2%, respectively) (Fig. 5I). Subtype-specific localization was most prominent in oligodendrogliomas, where 80.8% of *IDH1/2*-mutant/1p19q-co-deletion tumors originated in the frontal lobe compared to 32.1% of *IDH1/2*-wild-type GBM (Fig. S7). Based on the known associations between younger age and *IDH1/2*-mutant gliomas, we assessed whether age independently modulates anatomic–molecular correlations. Age stratification reinforced the observed patterns: 74.7% of frontal lobe gliomas in younger patients (<46 years) carried *IDH1/2* mutation, whereas 91.4% of temporal lobe and 100% of thalamic/basal ganglia tumors in older patients (≥46 years) belonged to the *IDH1/2*-wild-type glioma subtype (Fig. 5J).

Based on the association between anatomical localization and molecular subtypes, we further analyzed the high-frequency mutation profiles of gliomas in distinct neuroanatomical regions. Frontal lobe *IDH1/2*-mutant gliomas were characterized by frequent *TERTp* mutations (51.9%), *TP53* mutations (48.1%), 1p/19q co-deletion (45.5%), *CIC* mutations (34.2%), and *ATRX* mutations (31.2%) (Fig. 5K). Temporal lobe *IDH1/2*-wild-type GBM cases displayed a divergent profile dominated by *TERTp* mutations (80%), *EGFR* amplifications (44%), *PTEN* deletions (36%), *TP53* mutations (32%), and *CDKN2A/B* deletions (24%) (Fig. 5K). Thalamic–basal ganglia GBM cases exhibited recurrent chromosomal imbalance (chromosomal 7+/10–, 46.2%), *PTEN* deletions (41.2%), and lower frequencies of *TERTp* mutation (27.8%) and *EGFR* amplification (22.2%) (Fig. 5K). Kaplan–Meier survival curves showed significant differences across glioma subtypes (*P* < 0.0001; Fig. 5L). Because *TERTp* and *TP53* showed high-frequency mutations in gliomas, we conducted further subgroup analysis. The results showed that *TERTp* mutation is a poor prognostic factor in *IDH1/2*-wild-type gliomas, but a protective factor in *IDH1/2*-mutant gliomas (Fig. 5M). Notably, *TP53* mutation is a poor prognostic factor in gliomas and is not associated with the *IDH1/2* mutation status (Fig. 5M). Given the observed anatomic predilection of molecular subtypes, we further investigated whether spatial constraints of brain regions influence tumor growth. Tumor volume analysis demonstrated progressive reduction from frontal lobe to cerebellum/brainstem lesions, with larger tumors showing a correlation with a poor prognosis (Fig. S8).

## Discussion

This study systematically reveals the molecular alteration patterns and clinical translational value of gliomas through comprehensive integration of clinical characteristics, prognostic indicators, imaging features, and WES data from glioma patients. *IDH1/2* and *ATRX* gene mutations emerge as the early age-associated mutations in tumorigenesis, while molecular alterations such as *EGFR* amplification, *TERTp* mutations, and *PTEN* deletion predominantly occur during the later stages of disease progression. The WES-based molecular classification system revised the original diagnoses for approximately one-fourth of gliomas and revealed mutually exclusive patterns of genetic alterations along the tumorigenesis pathways. Additionally, based on the analysis of key tumor anatomical locations and molecular characteristics, this study found that gliomas carrying specific molecular variants exhibit anatomical preferences. These discoveries provide crucial insights for precision classification and therapeutic management of patients with gliomas.

This study demonstrated that a WES-based molecular classification system successfully revised 23.7% of original pathological diagnoses, a rate substantially higher than the 15% to 20% reclassification rate previously reported in single-center studies [11,12]. This discrepancy may be attributed to 2 key innovations. First, compared to targeted sequencing (limited to hotspot genes) commonly used in earlier single-center studies, WES enables systematic detection of rare mutations and structural variations. According to previous research, WES achieves 30% to 40% higher detection rates for *CDKN2A/B* homozygous deletions and *FGFR-TACC* fusions compared to targeted sequencing [13] , and these alterations serve as critical markers for revising glioma grades. Second, the inclusion of rare anatomic subtypes prone to misdiagnosis in conventional pathology (e.g., thalamic gliomas) revealed significant molecular heterogeneity compared to hemispheric gliomas [14,15]. Thalamic gliomas exhibit diagnostic error rates as high as 35% in traditional pathology because of their deep-seated location and biopsy-related challenges [16]. Thalamic gliomas exhibit diagnostic error rates as high as 35% in traditional pathology due to their deep-seated location and biopsy challenges [17], but is rare in supratentorial gliomas, frequently causing diagnostic discrepancies. These findings align with the 27.2% revision rate reported by Bi and colleagues [18] using TCGA dataset analyses, which further confirmed that molecular classification effectively overcomes the subjective limitations of histopathological examination. Notably, some histologically low-grade gliomas exhibit aggressive phenotypes due to *CDKN2A/B* homozygous deletions or *H3F3A* mutations [19], while certain high-grade tumors with *IDH1/2* mutations may benefit from low-intensity therapies [20]. Current neuro-oncology practice mandates molecular characterization, as it is critical for definitive histomolecular diagnosis and subsequent treatment planning, moving beyond an auxiliary role.

The present study systematically deciphered the complex patterns of pathway co-activation and mutual exclusivity in gliomas and revealed the unique nature of GBM as a “multi-pathway collaboratively driven tumor”. Compared to *IDH1/2*-mutant astrocytomas and oligodendrogliomas, GBM exhibits significantly higher pathway co-activation, with the p53 pathway and cell cycle dysregulation being the most predominant ones. The co-activation of p53 and cell cycle pathways in GBM (e.g., *TP53* mutations combined with *CDKN2A/B* deletions) may stem from synthetic lethality effects caused by DNA damage repair deficiencies. Previous studies have confirmed that, following p53 loss, *CDKN2A/B* deletion exacerbates genomic instability by inhibiting RB1 phosphorylation, thereby promoting tumor clonal evolution [21,22]. The hierarchical activation pattern of the RTK–PI3K–MAPK pathway in GBM likely promotes multi-pathway co-alterations through positive feedback loops. Our findings showed a positive correlation between *MET* mutations and *PIK3CA* alterations, consistent with a previous report: *MET* amplification enhances downstream AKT signaling through the activation of the IRS1-PI3K axis, while *PIK3CA* mutations further amplify this effect. Notably, *PIK3CA* mutations may confer resistance to MET inhibitors, supporting future clinical evaluation of combined PI3K and MET inhibitor therapies [23,24]. The mutual exclusivity of genetic alterations in the RTK pathway (e.g., EGFR/PDGFRA/MET/FGFR1) may reflect dosage sensitivity in signaling pathways [25]. TCGA analyses revealed that GBM cases harboring concurrent *EGFR* amplification and *PDGFRA* mutations are exceedingly rare [18], and tumor cells in vitro exhibit increased apoptosis due to extracellular signal-regulated kinase (ERK) hyperactivation [26]; these findings suggest that single RTK activation is sufficient to promote cell proliferation, whereas additional mutations may induce cytotoxicity. The mutual exclusivity between PI3K and MAPK pathway alterations (0.7% co-mutation rate) may induce conflicts in energy metabolic reprogramming.

We found that 73.4% of tumors exhibited unilobar confined growth, and patients with these tumors showed significantly prolonged OS compared to those with multilobar involvement. Muster et al. [27] revealed that 64% of *IDH1/2*-mutant gliomas showed unilobar growth compared to only 36% in *IDH1/2*-wild-type cases. A recent study reported a 5-year survival rate of 68% for frontal lobe *IDH1/2*-mutant tumors, which was markedly higher than that (12%) observed for multilobar *IDH1/2*-wild-type gliomas [28]. Beyond tumor biology, a critical contributing factor is the surgical advantage of unilobar tumors. Multilobar tumors frequently infiltrate functional areas (e.g., language or motor cortex), thereby hindering complete resection, with residual cells accelerating recurrence through epithelial–mesenchymal transition [29]. The strong frontal lobe preference of *IDH1/2*-mutant gliomas may be associated with their epigenetic features in OLIG2-positive precursor cells existing in this region, potentially driving selective expansion of *IDH1/2*-mutant clones through aberrant metabolic reprogramming [30–32]. IDH1/2-wild-type gliomas in elderly patients showed a marked anatomical specificity, with a high predilection for the temporal lobe and the thalamic–basal ganglia regions. The subventricular zone of the temporal lobe, which is rich in glial fibrillary acidic protein (GFAP)-positive neural stem cells with inherent EGFR signaling activity, may facilitate the accumulation of *IDH1/2*-wild-type mutations [33,34].

This study has several limitations. First, the retrospective design and dependence on existing cohorts may introduce selection bias, particularly due to underrepresentation of rare subtypes and non-Asian populations, which restricts the generalizability of conclusions. Second, a limited sample size in certain subgroups (e.g., thalamic GBM) reduced statistical power. Third, the cross-sectional survival analysis did not include longitudinal treatment response or glioma recurrence dynamics, which are critical for understanding resistance mechanisms. Fourth, the follow-up time for the patients was relatively short, and the right-censored events of patients were relatively high. Future research should focus on integrating tumor location with molecular characteristics and clinical outcomes using data from multi-center studies.

In conclusion, our study emphasized the necessity of integrating molecular, anatomical, and clinical data to refine glioma classification. Based on the molecular reclassification criteria, the final histopathologic classification was altered for approximately one-quarter of gliomas. On the basis of modern molecular criteria, we characterized the genomic diversity across different glioma subtypes. The prognosis of contemporary glioma patients was gradually improving. Glioma molecular subtypes correlate with preferential anatomical localization patterns. Molecular–anatomic correlations revealed subtype-specific spatial predilections.

## Materials and Methods

### Patient cohorts

This study enrolled 798 primary glioma patients from the single-center retrospective cohort at NFH between January 2019 and January 2024. The inclusion criteria were as follows: (a) histopathologically confirmed primary glioma, (b) availability of complete clinical and imaging data, and (c) no preoperative radiotherapy or chemotherapy. The exclusion criteria included pregnancy, presence of secondary gliomas, or concurrent malignancies. The study protocol was approved by the NFH Institutional Review Board (no. NFEC-2025-387). Written informed consent was obtained from all patients or their legal guardians.

### Clinical and imaging data collection

The following clinical data were collected: age, gender, clinical symptoms, extent of resection, histopathological grading (2021 WHO CNS5 criteria), and follow-up data. Imaging data were acquired using a 3-T MRI system, including T1-weighted contrast-enhanced and T2/fluid-attenuated inversion recovery (FLAIR) sequences. The anatomical localization of the tumor was independently assessed by 2 neuroradiologists, and discrepancies were resolved by a third senior reviewer. Tumor volume was semi-automatically segmented using ITK-SNAP (version 3.8), and nCBV was calculated with Olea Sphere (version 3.0).

### DNA extraction, library preparation, and genome sequencing

Formalin-fixed paraffin-embedded tumor slides or frozen fresh tumor tissues were collected along with paired blood samples. The sequencing was performed at the Simcere Diagnostics Laboratory (SimcereDx, Nanjing, China). Genomic DNA (gDNA) from tumor tissues was extracted using the Tissue Sample DNA Extraction Kit (Kai Shuo), while gDNA from paired leukocytes was extracted using MagMAX DNA Multi-Sample Ultra Kit (Thermo). The concentration of the extracted gDNA was quantified with the Qubit dsDNA HS Assay Kit by using a Qubit fluorometer (Thermo Fisher Scientific), and its quality was assessed using the Agilent 4200 TapeStation System. Next, 200 ng of the qualified gDNA was enzymatically fragmented into smaller pieces of 200 to 300 base pairs (bp) in size. The ends of these gDNA fragments were repaired with the VAHTS Universal Plus DNA Library Prep Kit (Vazyme Biotech, Nanjing, China; catalog no. ND617). The hybrid-enrichment-capture method was conducted using the KAPA HyperCapture Reagent Kit (Roche, USA). KAPA HyperChoice (Roche) and KAPA HyperExome (Roche) probes were used for the Neuro Onco 360 panel (SimcereDx) and WES, respectively, with the target size of 35.83 Mb. The manufacturer’s protocol included DNA fragmentation, repair with a-tailing (3′A-addition for DNA), adapter ligation, library enrichment and purification, library pooling, and hybrid-enrichment-capture library construction. Subsequently, the qualified DNA libraries were subjected to paired-end sequencing using the Illumina NovaSeq 6000 platform (Illumina, San Diego, CA, USA), and 150-bp reads were generated. The sequencing data for each sample were stored in FASTQ format. Subsequently, fastp software (version 2.20.0) was used to obtain various quality control metrics for each sample, trim adapters, and eliminate low-quality bases. The paired-end read sequences were aligned to the GRCh37 (hg19) reference genome using the Burrows-Wheeler Aligner algorithm (BWA-MEM version 0.7.17).

### Sequencing data analysis

The raw sequencing results were prepared as a FASTQ file (Illumina). The Bustard algorithm was used to convert the fluorescent signal into the actual sequence data, and the quality of the raw data was assessed using the Fast QC bioinformatics tool. Subsequently, a secondary analysis was conducted, in which the reads from the reference human genome (hg19) were compared, followed by post-alignment processing and calling of variants. The file format was then converted from FASTQ to BAM/SAM, and the results were output in a variant calling format file. Subsequently, a tertiary analysis was conducted and included annotations of variants; this analysis provided information to predict the functional impact of all variants established in the variant calling step. Subsequently, the single-nucleotide variation (SNV) and insertion/deletion (Indel) mutations were called and annotated using VarDict (version 1.5.7) and InterVar tools, respectively. SNVs and insertions/deletions (Indels) were called and annotated using VarDict (version 1.5.7). To ensure analysis consistency, all subsequent variant annotation and filtering steps were performed using resources specifically mapped to the GRCh37 build. The fusion genes were accessed through Factera (version 1.4.4), while the copy number variations (CNVs) were obtained using the CNV kit (dx1.1). The threshold for the minimal somatic variant allele frequency was set at 2%. The TMB was defined as the sum of nonsynonymous somatic mutations in the protein-coding region per megabase (muts/Mb).

Germline variants sequenced using paired blood samples were screened by VarDict (version 1.5.7) and then annotated to public databases, including gnomad (version 3.1.2), CLINVAR (202308), dbNSFP (version 42a), COSMIC (version 98), and the SimcereDx database. After filtration, the pathogenicity assessment for each mutation was performed according to the integrated outcomes of the InterVar and CLINVAR databases. According to the American College of Medical Genetics and Genomics guidelines, germline mutations were stratified into 5 categories.

### Statistical analysis

Categorical variables were compared using the chi-square test (or Fisher’s exact test when appropriate), and continuous variables were compared using the independent samples *t* test or the Mann–Whitney *U* test. Survival analysis was conducted based on Kaplan–Meier survival curves, with the log-rank test for intergroup differences (significance threshold: 2-sided *P* < 0.05). TMB was defined as nonsynonymous mutations per megabase and analyzed by linear regression for age correlation. All analyses were performed using R (version 4.3.1), GraphPad Prism (version 8.0), and SPSS (version 27.0).

## Data Availability

Any additional information required to reanalyze the data reported in this paper is available from the lead contact upon request (hgl1020@163.com).
