# Supplementary material for: Contemporary Precision Stratification and Prognostic Features of Primary Gliomas in a Southern Chinese Population
Source: Research (Wash D C). 2025 Dec 9;8:1014. doi: 10.34133/research.1014 (PMC12686340; doi:10.34133/research.1014)
Supplement: Supplementary 1 — Methods Figs. S1 to S8 Tables S1 to S3 [file research.1014.f1.docx]

Supplementary Materials

**This PDF file includes:**

Supplementary Text

Materials and Methods

Figs. S1 to S8

Tables S1 to S3

**Materials and Methods**

**Sequencing Platforms, Quality Control, and Sample Allocation**

All glioma samples and matched peripheral blood normal controls were subjected to DNA extraction. Whole-exome sequencing (WES) was the primary platform. For glioma samples with insufficient DNA yield or quality (DIN < 6.5 or quantity < 50 ng), a targeted sequencing approach was employed using the Neuro Onco 360 panel (SimeereDx, Nanjing, China) to capture key glioma-associated genes across a 35.83 Mb region. For WES, libraries were prepared using the VAHTS Universal Plus DNA Library Prep Kit and captured with the KAPA HyperExome probe set (Roche). Sequencing was performed on an Illumina NovaSeq 6000 platform with 150-bp paired-end reads. Samples were excluded from genomic analyses if they failed any of the following QC metrics: tumor purity < 20%, mean sequencing depth < 50x, or coverage (at 30x) < 90% of the target region. The flow chart is as follows.


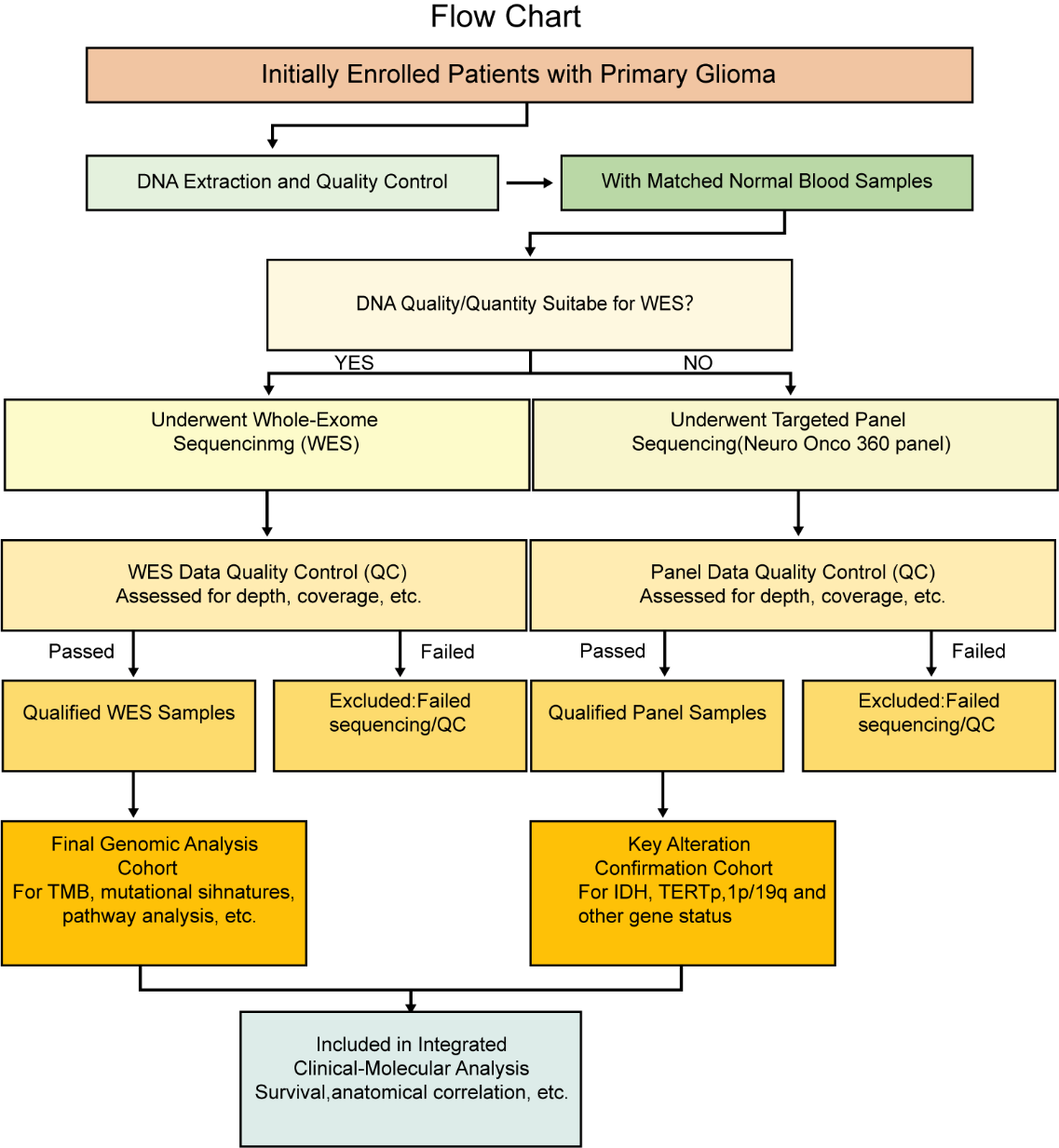


**Variant Annotation and Filtering**

Called variants were annotated and filtered using a suite of public databases. Population frequency filtering was performed against the Genome Aggregation Database (gnomAD v2.1.1), the 1000 Genomes Project (Phase 3, version 5), and the Exome Aggregation Consortium (ExAC v0.3.1) to exclude common germline polymorphisms. Functional and clinical impact was assessed using RefSeq gene models (2015-02-13 release), dbSNP (build 150), ClinVar (2023-08 release), and the catalogue of somatic mutations in cancer (COSMIC v92). The threshold for the minimal somatic variant allele frequency was set at 2%.

**Anatomical Localization Procedure of Glioma**

The primary anatomical lobe involvement for each tumor was independently assessed by two neuroradiologists, each with over 10 years of experience in neuro-oncologic imaging. Both raters were blinded to all molecular and clinical data. In cases of disagreement, a third senior neuroradiologist (with over 20 years of experience) independently reviewed the MRI scans. This arbitrator was also blinded to the initial assessments and all non-imaging data. The final anatomical classification was based on the arbitrator’s evaluation. Tumors were classified into predefined anatomical regions based on standard neuroanatomical atlases and widely accepted MRI landmarks, including the Sylvian fissure, central sulcus, and ventricular anatomy.

**DNA methylation analysis**

The methylation status of the MGMT promoter was determined using the MassARRAY EpiTYPER system (Agena Bioscience). Briefly, 140 ng of genomic DNA was subjected to bisulfite conversion using the EZ DNA Methylation Kit (Zymo Research) according to the manufacturer's instructions. This process deaminates unmethylated cytosines to uracils, while methylated cytosines remain unchanged. Bisulfite-converted DNA was then amplified by PCR using specific primers. The PCR products were treated with shrimp alkaline phosphatase (SAP) to dephosphorylate any remaining nucleotides. Subsequently, an in vitro transcription and base-specific cleavage reaction (MassCLEAVE) was performed using T7 RNA & DNA Polymerase and RNase A. This reaction generates a mixture of fragments whose masses are indicative of methylation status at individual CpG sites. The cleavage products were desalted using resin, spotted onto a SpectroCHIP, and analyzed by matrix-assisted laser desorption/ionization time-of-flight mass spectrometry (MALDI-TOF MS). The resulting spectra were analyzed, and methylation ratios were automatically quantified using EpiTYPER software (v1.0, Agena Bioscience).


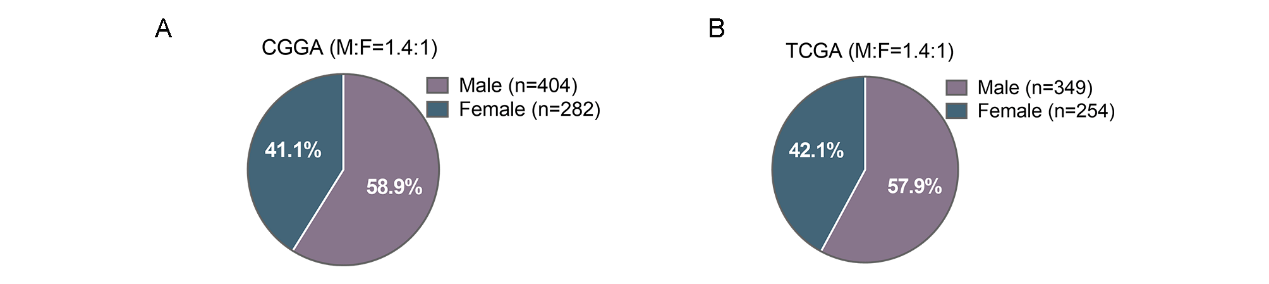


Fig. S1. The incidence of gliomas is higher in male. The ratio of male to female incidence is 1.4:1 in CGGA (A) and TCGA (B) cohorts.


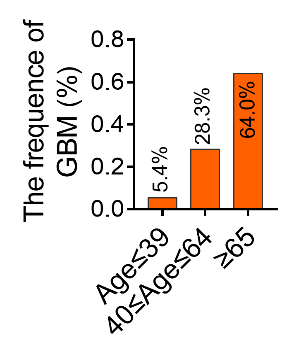


Fig. S2. The incidence of GBM increases with age in TCGA cohorts.


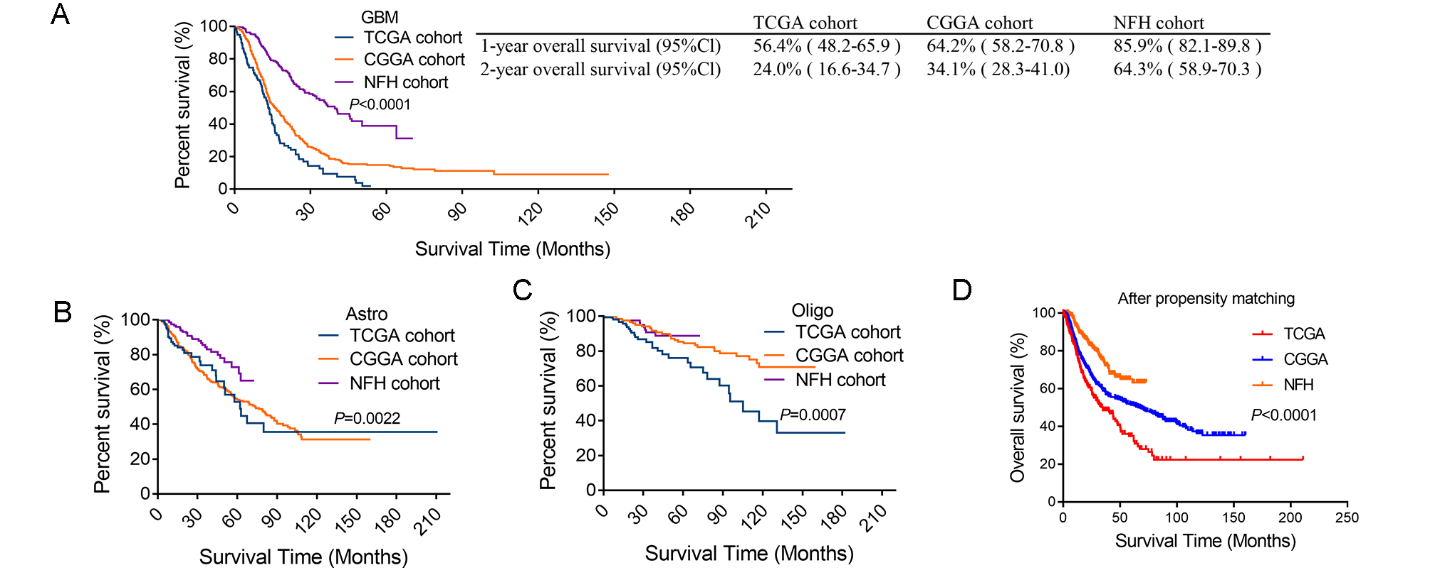


Fig. S3. The differential prognosis of glioma patients with different cohorts. (A-C) Kaplan-Meier analysis of patients with different histological subtypes in gliomas. (D) Patient survival curves in different cohorts after propensity score matching. Covariates included in the propensity model are: age, WHO grade, IDH status, 1p/19q status and radiotherapy/chemotherapy.


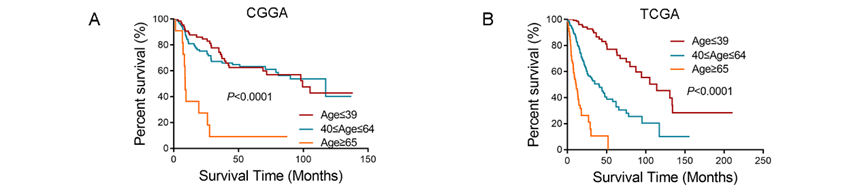


Fig. S4. Comparison of the prognosis among different age groups in gliomas. Advanced age was correlated with poor prognosis in CGGA (A) and TCGA (B) cohorts.


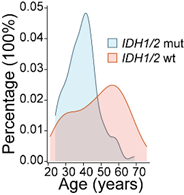


Fig. S5. The patients of CGGA cohort were divided into two categories according to IDH status. The peak age of onset in patients with IDH mutations is approximately 40 years, whereas for IDH wild-type patients, it occurs around 50 years.


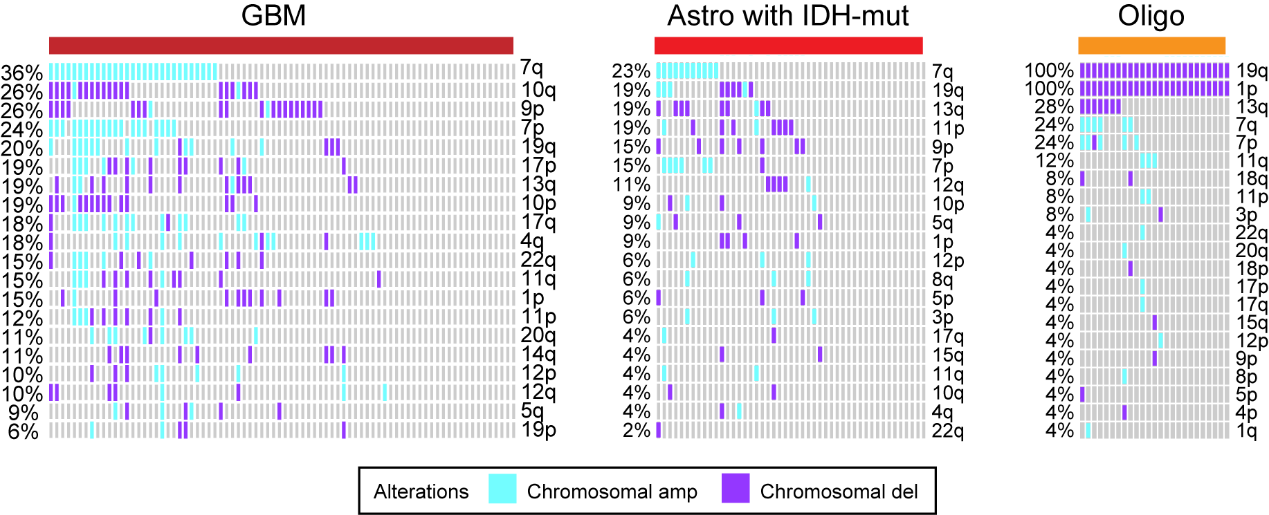


Fig. S6. Heatmap analysis of chromosomal alterations in glioma subtypes from the NFH cohort: glioblastoma (n=81), IDH-mutant astrocytoma (n=48), and oligodendroglioma (n=25).


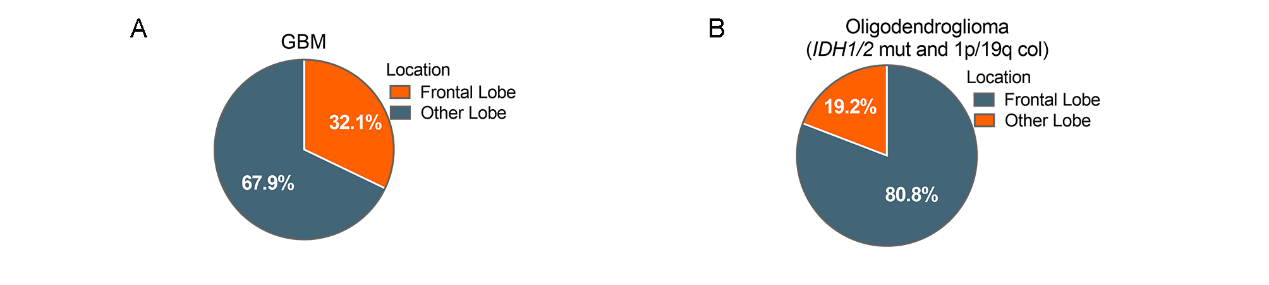


Fig. S7. Lobar distribution patterns of gliomas. (A) Glioblastoma (GBM) demonstrates frontal lobe involvement in only 32.1% of cases, whereas (B) oligodendroglioma shows predominant frontal lobe localization in 80.8% of cases.


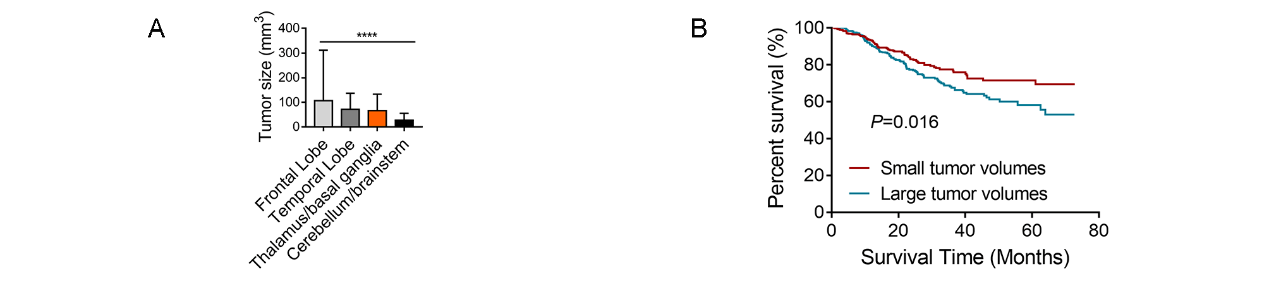


Fig. S8. Tumor volume across cerebral lobes and its prognostic impact. (A) Comparative analysis of tumor volume in different cerebral lobes; (B) Prognostic stratification based on tumor volume, with larger tumors demonstrating poorer clinical outcomes.

Table S1.

| **Table S1. Clinicopathological features of primary glioma patients in NFH cohort.** | |
| --- | --- |
| **Features** | **Cases (n=798)** |
| Age, years | 45.8±14.1 |
| Gender |  |
| Male | 480 (60.2%) |
| Female | 318 (39.8%) |
| Height (cm) | 165.0±7.6 |
| Body Mass Index (BMI, kg/m2) |  |
| BMI＜18.5 | 52 (6.5%) |
| 18.5≤BMI＜25 | 486 (60.9%) |
| BMI≥25 | 211 (26.4%) |
| NA | 49 (6.2%) |
| Karnofsky performance status |  |
| KPS＜70 | 45 (5.6%) |
| KPS≥70 | 753 (94.4%) |
| Headache symptom |  |
| Yes | 345 (43.2%) |
| No | 453 (56.8%) |
| Epilepsy |  |
| Yes | 195 (24.4%) |
| No | 603 (75.6%) |
| Tumor location |  |
| Supratentorial | 730 (91.5%) |
| Infratentorial | 68 (8.5%) |
| Contrast-enhanced T1-weighted imaging |  |
| Yes | 577 (72.3%) |
| No | 219 (27.4%) |
| NA | 2 (0.3%) |
| Increased intracranial pressure |  |
| Yes | 308 (38.6%) |
| No | 490 (61.4%) |
| Extent of resection |  |
| Biopsy | 2 (0.3%) |
| Partical resection | 75 (9.4%) |
| Subtotal and total resection | 721 (90.3%) |
| Original diagnosis |  |
| Glioblastoma | 364 (45.6%) |
| Astrocytoma | 185 (23.2%) |
| Oligodendroglioma | 141 (17.7%) |
| Others | 108 (13.5%) |
| *IDH1/2* status |  |
| Mutant | 314 (39.3%) |
| Wildtype | 484 (60.7%) |
| *1p/19q* status |  |
| Codeletion | 141 (17.7%) |
| Non-codeletion | 600 (75.2%) |
| NA | 57 (7.1%) |
| *MGMT* promoter methylation |  |
| Methylation | 150 (18.8%) |
| Non-methylation | 100 (12.5%) |
| NA | 548 (68.7%) |

Table S2. Univariate and multivariate Cox regression analysis for overall survival in glioma patients of NFH cohort.

|  | **Univariate Cox analysis** | | | **Multivariate Cox analysis** | | | |
| --- | --- | --- | --- | --- | --- | --- | --- |
| Features | HR | 95%CI | *P*-value | | HR | 95%CI | *P*-value |
| Age | 1.031 | 1.020-1.043 | <0.001 | | 1.008 | 0.996-1.021 | 0.198 |
| Gender | 0.775 | 0.568-1.058 | 0.108 | |  |  |  |
| Diagnoses | 0.514 | 0.427-0.619 | <0.001 | | 0.752 | 0.595-0.952 | 0.018 |
| WHO grade | 2.748 | 2.144-3.523 | <0.001 | | 3.073 | 2.079-4.543 | <0.001 |
| Tumor volume | 1.000 | 1.000-1.001 | 0.258 | |  |  |  |
| EOR | 0.684 | 0.442-1.060 | 0.089 | | 0.483 | 0.292-0.798 | 0.005 |
| Chemotherapy | 1.631 | 0.944-2.819 | 0.079 | | 7.984 | 1.963-32.477 | 0.004 |
| Radiotherapy | 1.955 | 1.158-3.300 | 0.012 | | 0.262 | 0.134-0.511 | <0.001 |

Abbreviations: HR, hazard ratio; EOR, extent of resection.

Table S3.

| **Table S3: Validated somatic mutants from exome sequencing of the gliomas.** | | | |
| --- | --- | --- | --- |
| **Gene** | **Accession** | **Amino Acid/ Promoter Change** | **Annotations** |
| *IDH1* | NM_005896.3 | p.R132H/p.R132G/p.R132S | >90% prevalence in *IDH*-mutant gliomas |
| *IDH2* | NM_005896.3 | p.R172K/p.R172G | *IDH2* mutations are observed in oligodendrogliomas |
| *ATRX* | NM_000489.4 | p.K1164fs/p.R2163^*^ | Truncating mutations cause loss of chromatin remodeling function |
| *TP53* | NM_000546.5 | p.R175H/p.R248Q/p.R273H/p.R282W | >80% prevalence in astrocytomas |
| *TERTp* | NM_198253.2 | C228T/C250T | Promoter mutations are common in GBM and oligo. |
| *PTEN* | NM_000267.3 | p.R130Q/p.R173C/p.R233^*^ | Truncating mutations are frequent in GBM |
| *MET* | NM_005228.3 | p.N375S/p.Y1253D | Amplification is predominant; point mutations are rare |
| *CIC* | NM_015125.4 | p.E1146K/p.Q1535^*^/p.R1510C | Associated with oligodendrogliomas |
| *FUBP1* | NM_000267.3 | p.R183C/p.R183H/p.R181C | Associated with oligodendrogliomas |
| *PIK3CA* | NM_006218.3 | p.H1047R/p.E545K/p.E542K | Activating mutations in the PI3K pathway |
| *NOTCH1* | NM_017617.4 | p.P2284S/p.L2483R/p.R2319H | Gain-of-function mutations in high-grade gliomas |
| *RB1* | NM_000321.2 | p.Q430^*^/p.R579fs | Predominantly truncating mutations |
| ***NF1*** | **NM_000267.3** | **p.R1276Q/p.R1391^*^/p.R681^*^** | **Associated with MAPK pathway activation** |
